# Structure–Property Relationship of Polybenzoxazine Composites for Advanced Applications

**DOI:** 10.3390/polym18151870

**Published:** 2026-07-30

**Authors:** Shakila Parveen Asrafali, Thirukumaran Periyasamy, Jaewoong Lee

**Affiliations:** Department of Fiber System Engineering, Yeungnam University, Gyeongsan 38541, Republic of Korea; thirukumaran@ynu.ac.kr (T.P.); jaewlee@yu.ac.kr (J.L.)

**Keywords:** polybenzoxazine, structure–property relationships, nanocomposites, fiber-reinforced composites, thermal properties and mechanical properties

## Abstract

Polybenzoxazines (PBz) represent a versatile class of high-performance thermosetting polymers that have attracted significant attention for advanced composite applications due to their unique combination of properties including high glass transition temperatures, low polymerization shrinkage, excellent thermal stability, and molecular design flexibility. This comprehensive review examines the structure–property relationships governing PBz composite performance, from molecular design principles through network formation, composite reinforcement strategies, and ultimate application performance. The review systematically addresses benzoxazine monomer structure and its influence on polymer network architecture, explores the polymerization mechanism, and critically evaluates composite design strategies incorporating carbon-based nanofillers, fiber reinforcements, and hybrid filler systems. Detailed analysis of structure–property relationships reveals how molecular and composite architecture control thermal stability (glass transition temperatures exceeding 350 °C and char yields up to 92%), mechanical performance, electrical properties (dielectric constants as low as 2.67), and chemical durability. Processing techniques ranging from conventional compression molding to emerging additive manufacturing approaches are discussed in the context of morphological control and property optimization. Applications spanning aerospace structures, high-frequency electronics and protective coatings demonstrate the technological relevance of PBz composites. Critical challenges including network brittleness, high cure temperatures, and recyclability limitations are addressed alongside recent advances in dynamic covalent networks, vitrimer chemistry, and self-healing systems that promise to overcome these barriers. This review provides a comprehensive framework for understanding and engineering polybenzoxazine composites for next-generation advanced applications.

## 1. Introduction

The development of advanced polymer composites for demanding applications in aerospace, electronics, energy, and protective systems requires materials that combine exceptional thermal stability, mechanical performance, chemical resistance, and processing flexibility. Traditional high-performance thermosetting resins such as epoxies, bismaleimides, and polyimides have dominated these applications for decades, yet each system presents inherent limitations in processing, property balance, or environmental sustainability [1,2,3,4,5]. Polybenzoxazines have emerged as a promising alternative platform that addresses many of these limitations while offering unprecedented molecular design flexibility. Polybenzoxazines are thermosetting polymers derived from the ring-opening polymerization of benzoxazine monomers, which are synthesized through a Mannich-type condensation reaction of phenols, primary amines, and formaldehyde [6,7,8,9,10,11,12]. The benzoxazine platform was first systematically developed in the 1990s, with pioneering work by Shen demonstrating the synthesis of benzoxazine and naphthoxazine monomers and their transformation into high-performance composites with processing windows around 260–290 °C, glass transition temperatures above 350 °C after postcuring, and exceptional char yields of 87–92% (after carbonization at 800 °C in N_2_ atm.) for optimized systems [13]. These early demonstrations established polybenzoxazines as viable candidates for applications requiring extreme thermal stability and structural performance.

The fundamental advantages of polybenzoxazines stem from their unique polymerization chemistry and resulting network structure. Unlike conventional phenolic resins that release water during cure and epoxies that undergo significant volumetric shrinkage, benzoxazine monomers polymerize through a thermally induced ring-opening mechanism that proceeds without the release of volatile byproducts and with minimal polymerization shrinkage [14,15,16,17,18,19]. This characteristic enables the fabrication of void-free composites with excellent dimensional stability and reduced internal stress. Furthermore, the resulting polybenzoxazine networks contain phenolic hydroxyl groups and Mannich bridge structures that provide extensive hydrogen bonding, contributing to high glass transition temperatures, excellent adhesion to reinforcements, and inherent flame retardancy through high char-forming tendency. The molecular design flexibility of benzoxazines represents perhaps their most distinctive feature. The three-component synthesis allows independent selection of phenol, amine, and aldehyde components, enabling systematic tuning of monomer structure and consequently polymer network properties. Electron-withdrawing or electron-donating substituents on the phenolic ring alter electronic polarizability and ring-opening kinetics, affecting dielectric properties and cure behavior. Bulky substituents and flexible linkages modify free volume and chain mobility, influencing glass transition temperature and mechanical properties. Functional groups can be introduced to enhance specific interactions with nanofillers or fibers, improving interfacial adhesion in composite systems [20,21,22,23]. This molecular tailorability has been exploited to design low-dielectric-constant benzoxazines for high-frequency electronics, bio-based benzoxazines from renewable phenolic feedstocks for sustainable applications, and dynamically crosslinked benzoxazines for recyclable and self-healing systems.

Comprehensive reviews by Kiskan et al. and Hamerton et al. have established polybenzoxazines as versatile phenolic-type resins with low viscosity, low curing shrinkage, and designable networks, providing foundational understanding of PBz chemistry, curing behavior, and structure–property relations [24,25]. These reviews position polybenzoxazines within the broader landscape of high-performance thermosets and highlight their comparative advantages and remaining challenges. The development of polybenzoxazine composites has progressed along several parallel and interconnected pathways [26,27,28,29,30]. Carbon-based nanocomposites incorporating graphene, carbon nanotubes, and reduced graphene oxide have been extensively investigated to enhance mechanical properties, thermal conductivity, electrical conductivity, and flame retardancy. Fiber-reinforced polybenzoxazine composites utilizing carbon fibers, glass fibers, and natural fibers have been developed for structural applications requiring high specific strength and stiffness. In addition to their excellent thermal and mechanical properties, benzoxazine resin composites possess remarkable ablation resistance. Quartz fiber-reinforced benzoxazine composites, benefiting from the synergistic effect of the thermally stable benzoxazine matrix and high-temperature-resistant quartz fibers, exhibit low ablation rates, high char retention, and excellent structural integrity under extreme thermal environments, making them promising materials for aerospace thermal protection systems, including radomes, rocket nozzles, and aerospace heat-shield structures. Hybrid composite systems combining multiple filler types or incorporating bio-based components have emerged to address sustainability concerns while maintaining performance [31,32,33,34,35,36]. Each composite design strategy requires careful consideration of filler dispersion, interfacial adhesion, processing compatibility, and the resulting structure–property relationships.

This review provides a comprehensive and critical examination of the structure–property relationships governing polybenzoxazine composite performance within the last 10 years. While earlier reviews mainly discuss benzoxazine chemistry, synthesis, and curing behavior, this article integrates molecular design, composite architecture, processing methods, and application performance within a unified framework. It critically compares carbon-based nanocomposites, fiber-reinforced composites, and hybrid systems, while also highlighting emerging topics such as sustainable composites, recyclable/vitrimer-like networks, and self-healing materials. This comprehensive structure–property–application perspective provides a broader and more application-oriented understanding than existing reviews. Section 2 addresses molecular design principles and polymerization mechanisms. Section 3 examines composite design strategies including carbon-based nanocomposites, fiber-reinforced systems, and hybrid composites. Section 4 analyzes structure–property relationships for thermal, mechanical, electrical, and chemical resistance properties. Section 5 discusses processing techniques and morphological control. Section 6 surveys applications across aerospace, electronics, coatings, and energy systems. Section 7 addresses current challenges and future research directions, including recyclability and self-healing systems. Section 8 provides concluding remarks and perspectives on the future of polybenzoxazine composites for advanced applications.

## 2. Molecular Design and Polymer Network Formation

### 2.1. Benzoxazine Monomer Structure Based on Aromatic and Aliphatic Reactants

The molecular structure of benzoxazine monomers is the primary determinant of polybenzoxazine network properties, and the three-component synthesis provides exceptional flexibility for property tuning. Benzoxazine monomers are synthesized through Mannich condensation of a phenolic compound, a primary amine, and formaldehyde, forming a six-membered heterocyclic ring containing oxygen and nitrogen. The general structure consists of a benzene ring fused to an oxazine ring, with the phenolic and amine components providing sites for structural variation [37,38,39,40,41]. The choice of phenol determines the electronic properties of the aromatic ring, the availability of reactive sites, and the potential for hydrogen bonding in the cured network. The amine component influences the flexibility, polarity, and steric characteristics of the resulting monomer and polymer. This synthetic flexibility enables systematic exploration of structure–property relationships through rational monomer design.

**Figure 1:**
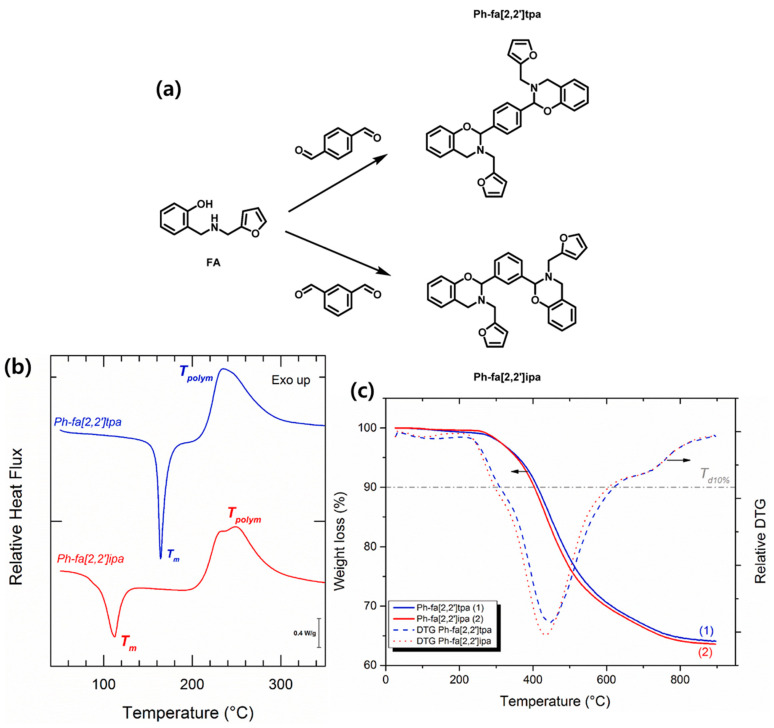
(**a**) Schematic representation showing the synthesis of aromatic dialdehyde-based benzoxazines from terepthaldehyde and isophthalaldehyde; (**b**) DSC of Ph-fa[2,2′]ipa and Ph-fa[2,2′]tpa at 10 °C·min^−1^; and (**c**) TGA of cured materials under nitrogen atmosphere. Reproduced with permission from [6].

Tavernier et al. [6] examined the influence of isomerism on the thermal properties of two aromatic dialdehyde-based bisbenzoxazines, based on terepthaldehyde (para configuration) and isophthalaldehyde (meta configuration) as shown in Figure 1. Both monomers formed rapidly at room temperature within a few minutes. Product isolation was simplified by selecting reaction solvents in which the target monomers were insoluble. Initially, reactions were conducted in refluxing toluene; however, only Ph-fa[2,2′]tpa precipitated, whereas Ph-fa[2,2′]ipa remained soluble. Therefore, the reaction conditions were optimized using acetone for Ph-fa[2,2′]tpa and ethanol for Ph-fa[2,2′]ipa, allowing the products to precipitate directly from the reaction medium and be collected by simple filtration. DSC analysis showed that Ph-fa[2,2′]tpa exhibited a sharp melting endotherm at 164 °C, whereas Ph-fa[2,2′]ipa melted at 112 °C with a broader peak, indicating lower crystallinity due to its meta-isomeric structure (Figure 1b). Both monomers underwent thermal polymerization with similar onset temperatures (216 and 212 °C) and peak maxima (235 and 249 °C) for Ph-fa[2,2′]tpa and Ph-fa[2,2′]ipa, respectively, reflecting their comparable oxazine ring structures. The polymerization enthalpies were also similar (257 and 278 J g^−1^). However, Ph-fa[2,2′]tpa showed a broad exotherm with a slight shoulder, while Ph-fa[2,2′]ipa exhibited a bimodal peak, likely arising from the presence of two diastereomers with slightly different curing behaviors. Both poly(Ph-fa[2,2′]tpa) and poly(Ph-fa[2,2′]ipa) exhibited similar thermal stability, with Td_10_% values of 412 and 401 °C, respectively, and identical char yields of 64% at 900 °C (Figure 1c). DTG analysis showed comparable degradation profiles, with maximum weight loss at 447 °C for poly(Ph-fa[2,2′]tpa) and 436 °C for poly(Ph-fa[2,2′]ipa). A minor shoulder around 720 °C was observed for both polymers, likely associated with the release of volatile species during char formation. These results demonstrate that while structural conformation can effectively improve monomer processability, it has little effect on polymerization and degradation behavior [42].

**Figure 2:**
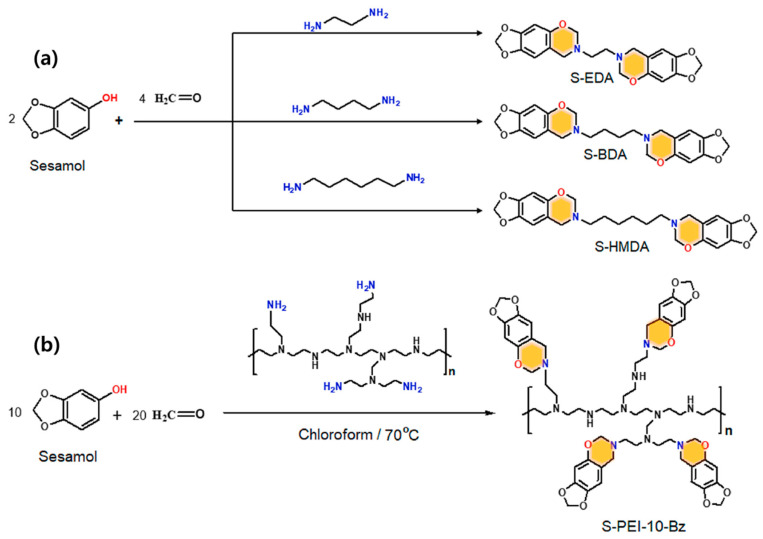
The synthesis route for (**a**) sesamol diamine-based benzoxazine monomers; and (**b**) sesamol-PEI-based benzoxazine resins. Reproduced with permission from [43].

Necolau et al. [43] first explored the grafting of benzoxazine rings onto branched polyethyleneimine (PEI) using sesamol via Mannich condensation (Figure 2). Sesamol-based benzoxazine monomers (S-EDA, S-BDA, and S-HMDA) were synthesized by reacting sesamol with paraformaldehyde and the corresponding diamines in chloroform under reflux at 70 °C for 8 h. The products were purified by washing with NaOH and water, dried over MgSO_4_, and isolated as yellow solids. PEI-based benzoxazine resins were prepared similarly by reacting sesamol, PEI (Mw ≈ 800 g mol^−1^), and paraformaldehyde in chloroform under reflux for 8 h. The products were washed, dried, and concentrated under reduced pressure to obtain brown-yellow viscous resins. The study showed that benzoxazine formation, crosslink density, and thermoset properties were strongly influenced by the phenolic substituent and amine reactivity. The polymerization temperature and curing enthalpy varied with the diamine structure. Polymerization temperature increased with aliphatic chain length, while S-EDA exhibited the lowest onset temperature of 186 °C, due to its shorter spacer. Sesamol-PEI-based systems exhibited relatively low benzoxazine grafting (~15%) but displayed lower curing temperatures (179 and 222 °C), higher glass transition temperatures (>129 °C), improved char yields (41–44%), and enhanced flame-retardant properties (LOI = 33–43), suggesting the methylenedioxy group of sesamol contributes to crosslinking. Mechanical performance was closely related to the degree of benzoxazine functionalization, with higher aromatic oxazine content leading to increased modulus and hardness [44,45,46,47]. The results suggest that sesamol’s methylenedioxy group may contribute to additional crosslinking, improving thermal and mechanical performance despite slightly lower thermal stability.

### 2.2. Polymerisation Mechanism and Network Structure

The polymerization of benzoxazine monomers proceeds through a thermally induced ring-opening mechanism that is fundamentally different from the condensation polymerization of conventional phenolic resins or the addition polymerization of epoxies. Upon heating, the oxazine ring undergoes thermal ring-opening to generate a reactive iminium ion intermediate and a phenoxide ion. These reactive species then participate in electrophilic aromatic substitution reactions, with the iminium ion attacking the ortho or para positions of phenolic rings on neighboring molecules. This process forms Mannich bridge linkages (–CH_2_–NR–CH_2_–) connecting aromatic rings and generates phenolic hydroxyl groups. The polymerization is autocatalytic, with the generated phenolic hydroxyl groups accelerating further ring-opening through hydrogen bonding interactions with unreacted oxazine rings. A key structural feature of polymerizable benzoxazines is the position of the oxazine ring relative to the phenolic moiety, which enables polymerization. Among benzoxazine isomers, only 1,3-benzoxazines undergo ring-opening polymerization (ROP) because of the unique conformation of their heterocyclic ring. Numerous studies have investigated how monomer structure influences the polymerization behavior and resulting thermoset properties [48,49,50,51].

The ring-opening polymerization mechanism has several important consequences for composite processing and properties. First, the polymerization proceeds without the release of volatile byproducts, in contrast to phenolic resins that release water during cure. This characteristic enables the fabrication of void-free composites with excellent dimensional stability. Second, the polymerization exhibits minimal volumetric shrinkage, typically less than 1%, compared to 5–10% for epoxy resins. This low shrinkage reduces residual stress in composites and improves dimensional accuracy. Third, the autocatalytic nature of the polymerization means that cure kinetics are sensitive to temperature history, monomer structure, and the presence of catalysts or accelerators. Understanding and controlling cure kinetics is essential for optimizing processing conditions and achieving desired network structures.

**Figure 3:**
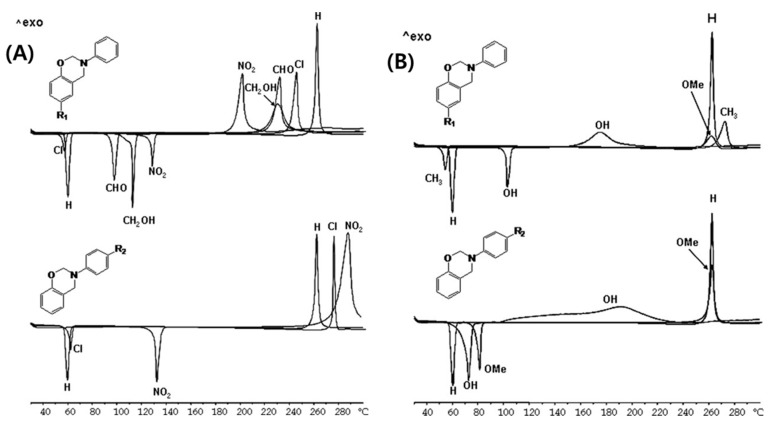
DSC plots of benzoxazine monomers with (**A**) electron-withdrawing groups in the 6 and 4′ positions; and (**B**) electron-donating groups in the 6 and 4′ positions. Reproduced with permission from [20].

Monomer structure strongly influences the polymerization behavior and properties of benzoxazines. Andreu et al. [20] studied the effect of electron-withdrawing groups (EWGs) and electron-donating groups (EDGs) on the ring-opening polymerization (ROP) temperature of benzoxazines. DSC analysis, represented in Figure 3A, showed that benzoxazines bearing electron-withdrawing substituents at the 6-position exhibited significantly lower polymerization temperatures, with the effect increasing alongside substituent electron-withdrawing strength. This behavior is attributed to enhanced phenol acidity, which promotes autocatalytic ring-opening polymerization. In contrast, electron-withdrawing substituents at the para-position of the phenyl ring increased the polymerization temperature by destabilizing the protonated iminium intermediates involved in the propagation step. Notably, the hydroxymethyl-substituted monomer polymerized at an unusually low temperature, suggesting a distinct polymerization mechanism. Whereas electron-donating substituents had little effect on the thermal polymerization of benzoxazines, irrespective of their position, except for hydroxyl-substituted monomers, which exhibited markedly lower polymerization temperatures. The phenolic hydroxyl group catalyzed ring-opening polymerization, initiating the reaction at temperatures as low as 100 °C. Its strong activating effect also introduced additional propagation pathways, producing highly crosslinked, brittle polymer networks with no detectable glass transition.

**Figure 4:**
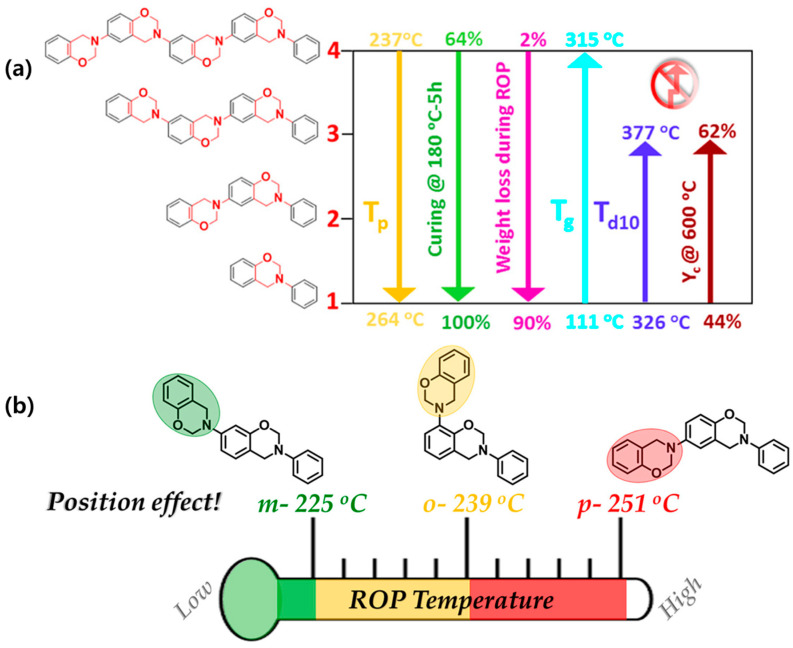
Role of (**a**) number of oxazine rings in the benzoxazine backbone and their thermal characteristics; and (**b**) position of oxazine ring in the benzoxazine backbone on their ring-opening polymerization. Reproduced with permission from [48,52].

Kolanadiyil et al. [52] showed that increasing the number of oxazine rings per monomer reduces polymerization temperatures, enhances thermal stability, and limits volatilization during curing, although longer curing times may be required (Figure 4a). Bisbenzoxazines are generally preferred because they provide higher crosslink densities than monobenzoxazines. The relative position of oxazine rings also affects reactivity. Kolanadiyil et al. [48] reported that ortho-, meta-, and para-substituted bisbenzoxazines exhibit different ROP behaviors due to variations in intra- and intermolecular interactions, as shown in Figure 4b. The meta configuration showed the highest reactivity, attributed to favorable hydrogen bonding and intramolecular catalytic effects. Similarly, Liu and Ishida [11] found that bisphenol F isomerism significantly influences thermoset properties. Unexpectedly, the ortho–ortho isomer produced polybenzoxazines with higher glass transition temperatures, crosslink densities, and thermal stability than the para-containing isomers, emphasizing the critical role of molecular design in tailoring material performance.

Cure schedules and post-curing treatments play a critical role in determining the crosslink density and glass transition temperature (T_g_) of polybenzoxazines. Shen et al. [13] demonstrated that optimized curing at 260–290 °C can produce T_g_ values exceeding 350 °C in naphthoxazine systems. Incomplete curing leads to residual monomer and lower crosslink density, while excessive curing may cause thermal degradation and loss of network integrity. Network structure is also influenced by heterogeneity arising from incomplete cure, phase separation, and variations in crosslink density. Blends of mono- and bifunctional benzoxazines can form soft and hard domains with distinct thermal and mechanical properties, enabling a balance between stiffness and toughness. In addition, the phenolic hydroxyl groups generated during polymerization form extensive hydrogen-bonding networks that act as physical crosslinks, enhancing T_g_, mechanical performance, and adhesion. Although measuring crosslink density in polybenzoxazines is challenging due to their complex network architecture and strong hydrogen bonding, it remains a key factor governing thermal stability, modulus, and overall material performance. Molecular design and curing conditions therefore provide effective tools for tailoring network properties.

## 3. Composite Design and Reinforcement Strategies

### 3.1. PBz Nanocomposites with Carbon-Based Nanofillers

Carbon-based nanofillers including graphene, carbon nanotubes, and reduced graphene oxide have been extensively investigated as reinforcements for polybenzoxazine matrices due to their exceptional mechanical properties, electrical and thermal conductivity, and high aspect ratios that enable efficient load transfer and property enhancement at low loadings. The incorporation of carbon nanofillers into polybenzoxazine matrices addresses several limitations of the neat polymer including brittleness, limited electrical conductivity, and modest thermal conductivity, while potentially enhancing flame retardancy through char formation synergies. The success of carbon-based nanocomposites depends critically on achieving uniform filler dispersion, strong interfacial adhesion between filler and matrix, and control of filler orientation and network formation.

**Figure 5:**
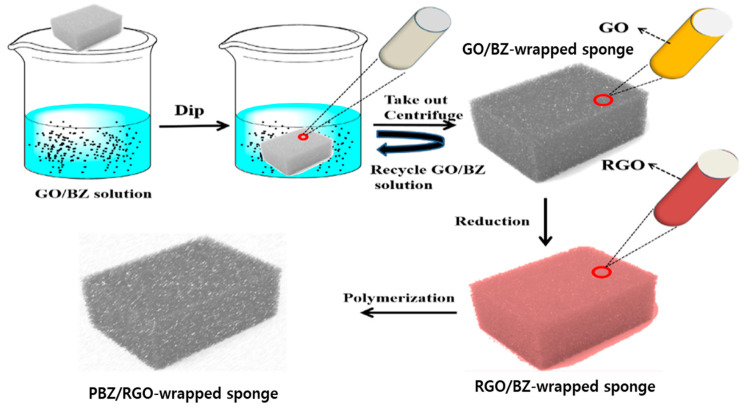
Schematic representation showing the preparation of polybenzoxazine/reduced graphene oxide-coated cellulose sponge (PBZ/RGO-CS). Reproduced with permission from [53].

Qin et al. [53] developed a multifunctional polybenzoxazine/reduced graphene oxide-coated cellulose sponge (PBZ/RGO-CS) with superhydrophobic/superoleophilic properties for efficient oil–water separation, as shown in Figure 5. CS was selected as a low-cost, renewable, and eco-friendly substrate. BA-a benzoxazine monomer was synthesized via the Mannich reaction. GO was dispersed in DMF by ultrasonication for 12 h, followed by the addition of a 5 wt.% benzoxazine solution. CS was impregnated with the GO/benzoxazine suspension through three dipping–squeezing cycles, coated GO was reduced with HI, vacuum dried at 60 °C for 2 h, and stepwise cured at 100, 120, and 140 °C (1 h each) to obtain the composites.

Polybenzoxazine effectively anchored reduced graphene oxide onto the CS framework, increasing the water contact angle from 0° for pristine CS to 155° for PBZRGOS5. The enhanced surface roughness with increasing RGO loading transformed the surface from superhydrophilic to superhydrophobic. The interconnected GO network significantly enhanced the mechanical stability of the PBZRGOS composite. PBZRGOS5 exhibited excellent elastic recovery after repeated compression, retaining its structure with minimal plastic deformation even after 100 cycles, demonstrating outstanding durability and resilience. The strong hydrophobic and π–π interactions between RGO and PBZ further reinforced the 3D porous framework. The PBZ coating reduced surface energy and acted as a binder for RGO, producing a mechanically robust material with excellent separation efficiency (99.1%) and high oil absorption capacity (66–123 g/g), depending on liquid density. Adsorption capacity showed an approximately linear relationship with liquid density, indicating that absorption performance was primarily governed by the density of the absorbed liquid. Under sunlight, the RGO layer converted solar energy into heat, reducing crude oil viscosity and significantly enhancing oil uptake [54,55]. The sponge maintained high performance after repeated mechanical cycling and separation tests, demonstrating its potential as a low-cost, durable, and energy-efficient material for oil spill remediation and oily wastewater treatment.

### 3.2. PBz Composites from Carbon Fiber Reinforced with PBz-Based Vitrimers

Fiber-reinforced polybenzoxazine composites are among the most advanced applications of polybenzoxazines in structural materials, combining the high specific strength and stiffness of continuous fibers with the excellent thermal stability, low shrinkage, and flame resistance of polybenzoxazine matrices. Carbon fiber-reinforced polymer (CFRP) composites are widely employed in aerospace and satellite structures because of their outstanding strength-to-weight ratio, enabling lightweight and fuel-efficient designs. However, conventional epoxy-based CFRPs can suffer degradation in low Earth orbit (LEO) due to atomic oxygen, radiation, vacuum exposure, thermal cycling, and micrometeoroid impacts, which may reduce mechanical performance and promote delamination. As a result, alternative matrices such as polyimides, cyanate esters, and particularly polybenzoxazines (PBZs) have gained increasing attention. Owing to their superior thermal stability, environmental resistance, low flammability, and potential radiation-shielding capability, polybenzoxazine-based CFRPs are promising candidates for high-performance and durable space applications, including deployable structures subjected to elevated strains [31,56,57].

**Figure 6:**
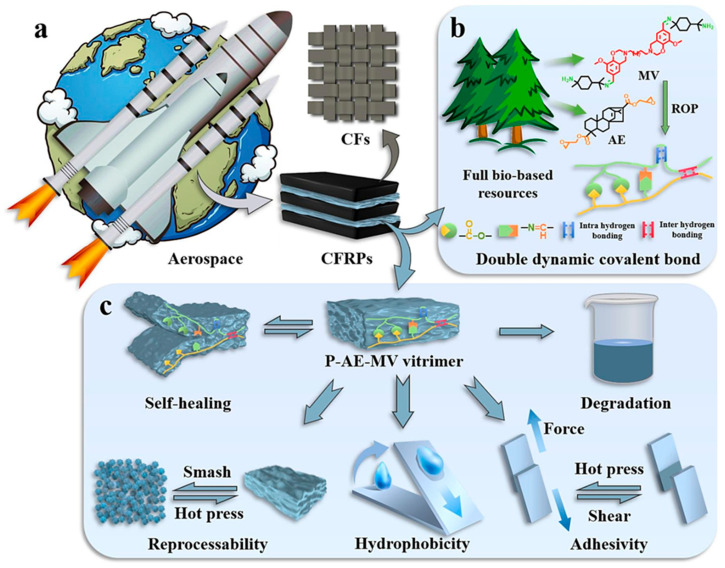
(**a**) The application of carbon fiber-reinforced polymers (CFRPs) in aerospace; (**b**) preparation of double dynamic covalent bond vitrimer network from full bio-based raw materials; and (**c**) self-healing, degradation, reprocessability, hydrophobicity and adhesivity of P-AE-MV vitrimers. Reproduced with permission from [1].

Zhou et al. (2024) [1] developed a bio-based polybenzoxazine vitrimer matrix for carbon fiber-reinforced polymers (CFRPs) using vanillin-derived benzoxazine and rosin-based epoxy resin (Figure 6). Vitrimers are an emerging class of polymeric materials containing dynamic covalent bonds capable of reversible bond exchange, enabling unique properties such as self-healing, shape memory, reprocessability, and controlled degradation. Common dynamic chemistries employed in vitrimer design include ester, carbamate, Schiff base, and disulfide linkages [58,59,60,61]. In this study, a fully bio-based benzoxazine monomer (VD) was synthesized from vanillin and 1,10-diaminodecane and subsequently reacted with excess 1,8-menthane diamine (MDA) to form a Schiff base curing agent (MV). The resulting MV was used to cure acrylpimaric acid diglycidyl ester (AE), producing a catalyst-free vitrimer matrix (P-AE-MV) containing dual dynamic covalent networks based on Schiff base and β-hydroxy ester linkages. The incorporation of benzoxazine structures significantly enhanced the mechanical strength, thermal stability, char yield, and hydrophobicity of the vitrimer matrix. The improved tensile strength was attributed to increased ring-opening reactions of epoxy and benzoxazine groups, generating additional hydroxyl functionalities and consequently a denser intermolecular hydrogen-bonding network. Owing to the synergistic effect of the dual dynamic covalent bonds and the autocatalytic action of tertiary amines generated during curing, P-AE-MV exhibited a low activation energy for bond exchange (E_a_ = 48.1 kJ mol^−1^), facilitating rapid network rearrangement.

As a result, the vitrimer displayed excellent self-healing, shape-memory, reprocessing, and degradability characteristics. The self-healing efficiency reached 98.2% at 160 °C, while the second-cycle reprocessed sample retained 82.7% of its original mechanical performance. Carbon-fiber-reinforced composites (P-AE-MV-CF) fabricated using this matrix demonstrated outstanding mechanical properties, self-adhesion, shape-memory behavior, and recyclability due to dynamic Schiff base and β-hydroxy ester bonds [62,63,64]. Notably, complete matrix degradation in n-butylamine at 60 °C within 30 min enabled efficient and non-destructive recovery of carbon fibers. These findings highlight a sustainable strategy for developing high-performance, recyclable CFRPs from renewable resources, thereby promoting closed-loop carbon-fiber recycling and expanding the application of polybenzoxazine-based vitrimers in advanced composite materials.

**Figure 7:**
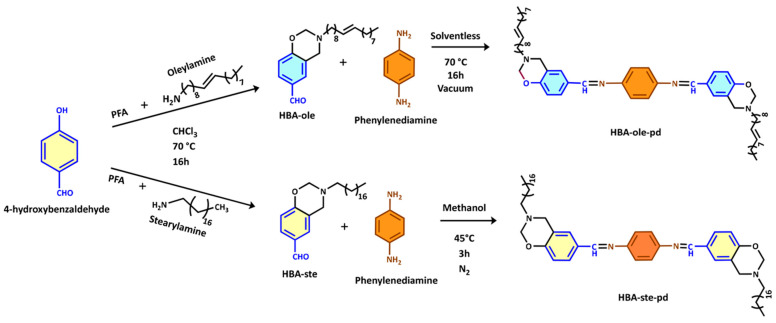
Synthesis routes for obtaining two different Schiff base functionalized benzoxazine monomers: HBA-ste-pd and HBA-ole-pd. Reproduced with permission from [56].

Rai et al. [56] investigated polybenzoxazine-based vitrimers containing dynamic imine bonds and demonstrated that amine unsaturation significantly influences vitrimer performance (Figure 7). Compared with stearylamine-derived systems, oleylamine-based polybenzoxazines exhibited faster stress relaxation, lower activation energy for bond exchange, higher crosslink density, and improved self-healing and reprocessability. The study highlights the advantages of using oleylamine-derived polybenzoxazine vitrimer, p(HBA-ole-pd), as a matrix for reprocessable composites. The oleylamine double bond improves monomer processability, fibre–matrix adhesion, and crosslink density, resulting in enhanced thermal stability (T_10%_ = 312 °C), lower activation energy for imine exchange (61.35 kJ mol^−1^), and faster stress relaxation [with a relaxation time (τ) of 50 s at 140 °C in p(HBA-ole-pd) compared to 122 s in p(HBA-ste-pd)] compared to the stearylamine-based counterpart. Dynamic imine linkages enabled efficient self-healing, reprocessing, and recycling, with recovered composites retaining mechanical properties comparable to the original materials [65,66,67,68]. Furthermore, the matrix could be selectively degraded during recycling without damaging the carbon fibres, allowing their complete recovery and reuse. Carbon fiber-reinforced composites prepared from the oleylamine-based vitrimer showed excellent mechanical properties and closed-loop recyclability, enabling complete carbon fiber recovery and reuse under mild chemical conditions. The study highlights the potential of unsaturated polybenzoxazine vitrimers for sustainable, high-performance recyclable composites.

### 3.3. Hybrid PBz Composites Containing Nanofillers and Fibers

Hybrid composite systems incorporating multiple filler types or combining nanofillers with fiber reinforcements represent an advanced approach to polybenzoxazine composite design, enabling synergistic property enhancement and multifunctionality. Hybrid composites can address multiple performance requirements simultaneously, such as combining mechanical reinforcement with electrical conductivity, thermal management, or flame retardancy. The design of hybrid composites requires careful consideration of filler compatibility, processing complexity, and potential antagonistic interactions between different fillers [69,70,71,72,73]. Inorganic nanofillers including silica, bio-silica, and polyhedral oligomeric silsesquioxane (POSS) have been incorporated into polybenzoxazine matrices to enhance thermal stability, flame retardancy, and mechanical properties. Bio-silica derived from rice husk ash or other agricultural waste has been incorporated into bio-based polybenzoxazines for improved thermal stability and for self-healing and vitrimeric composite applications. The high surface area and reactive surface hydroxyl groups of silica nanoparticles enable strong interfacial interactions with polybenzoxazine matrices through hydrogen bonding and potential covalent bonding during cure. Silica nanofillers can also improve flame retardancy by forming protective surface layers during combustion and by diluting the fuel concentration in the composite.

**Figure 8:**
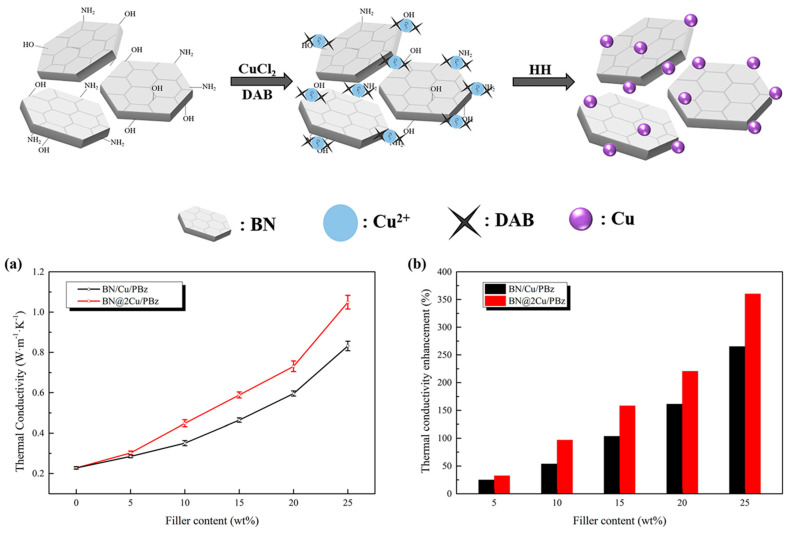
(**Top**) Schematic representation showing the synthesis of the BN@Cu hybrid filler and (**Bottom**) (**a**) λ values of the BN@2Cu/PBz and BN/Cu/PBz composites as a function of filler content; (**b**) enhancement in thermal conductivity of BN@2Cu/PBz and BN/Cu/PBz composites. Reproduced with permission from [74].

Wang et al. [74] developed a polybenzoxazine composite containing a hybrid boron nitride–copper (BN@Cu) filler, in which Cu nanoparticles were deposited onto BN flakes to create a hierarchical thermally conductive network. Hexagonal boron nitride (h-BN) was dispersed in 0.1 M CuCl_2_ solution, followed by the sequential addition of p-phenylenediamine (DAB), NH_3_·H_2_O (pH 10), hydrazine hydrate, and PVP to synthesize BN@Cu hybrid fillers, which were then filtered, washed, and vacuum-dried. BN@Cu and BOZ were ball-milled for 1 h and hot-pressed at 12 MPa using a stepwise curing schedule (160–220 °C, 1 h at each temperature). During synthesis, DBA promoted Cu^2+^ adsorption onto h-BN, while hydrazine reduced Cu^2+^ to metallic Cu, enabling the nucleation and growth of Cu nanoparticles on the BN surface. Pure PBz exhibited a low thermal conductivity (λ) of 0.228 W m^−1^ K^−1^. Compared with BN/Cu blends, BN@2Cu hybrid fillers consistently achieved higher λ values at all filler loadings, reaching 1.049 W m^−1^ K^−1^ at 25 wt.%, compared with 0.832 W m^−1^ K^−1^ for BN/Cu (Figure 8). The superior performance is attributed to the dot–plane structure of BN@2Cu, which stabilizes thermally conductive networks, prevents BN restacking, and reduces filler–matrix interfaces, thereby suppressing phonon scattering and enhancing heat transfer [75,76,77,78,79]. Despite the addition of Cu, the composites maintained low dielectric constant and loss values, as well as excellent electrical insulation, making them promising materials for thermal management applications.

Kong et al. [57] investigated ultrathin carbon- and Kevlar^®^-reinforced polybenzoxazine laminates containing 5–10 wt.% polyhedral oligomeric silsesquioxane (POSS) under simulated low Earth orbit (LEO) conditions. POSS significantly improved resistance to atomic oxygen erosion by forming a protective silica-rich surface layer, reducing surface roughness and increasing thermal stability. Laminates containing 10 wt.% POSS showed the best retention of glass transition temperature, flexural strength, and stiffness after up to 18 months of simulated exposure. While gamma radiation and vacuum ultraviolet (VUV) exposure also caused degradation, POSS-modified composites consistently outperformed unmodified laminates, demonstrating their potential for durable deployable space structures.

## 4. Structure–Property Relationships

### 4.1. Thermal Properties

Thermal properties such as glass transition temperature (T_g_), thermal stability, thermal expansion, and thermal conductivity are key factors governing the performance of polybenzoxazine composites in high-temperature applications. Polybenzoxazines are known for their exceptionally high T_g_ values, often exceeding 350 °C after optimized curing and post-curing, owing to their high crosslink density, rigid aromatic structure, and extensive hydrogen-bonding network [13,22]. T_g_ is primarily controlled by crosslink density, hydrogen bonding, and curing conditions. Bifunctional monomers and optimized cure schedules increase network rigidity and T_g_, while flexible segments or plasticizing additives generally reduce it. Fillers such as graphene, carbon nanotubes, silica, and carbon fibers can further enhance T_g_ by restricting polymer chain mobility and strengthening interfacial interactions.

Polybenzoxazines also exhibit excellent thermal stability, with decomposition temperatures typically above 300–400 °C and char yields of 50–60% in nitrogen, which can exceed 80% in specially designed systems. Thermal stability is enhanced by high aromatic content, crosslink density, and the incorporation of heteroatoms such as nitrogen or silicon. Nanofillers and inorganic additives further improve stability by promoting char formation and acting as thermal barriers. In addition, polybenzoxazine composites offer moderate coefficients of thermal expansion (CTE), which can be significantly reduced through reinforcement with carbon fibers or other low-CTE fillers, improving dimensional stability in demanding environments. These combined characteristics make polybenzoxazine composites attractive candidates for aerospace, electronics, and other high-temperature applications.

### 4.2. Mechanical Properties

Mechanical properties such as modulus, strength, toughness, and fatigue resistance are critical for structural applications of polybenzoxazine composites. Owing to their rigid aromatic backbone, high crosslink density, and extensive hydrogen bonding, polybenzoxazines exhibit high stiffness and strength, with neat resins typically showing moduli of 3–4 GPa [22]. However, their highly crosslinked network also leads to brittleness and limited fracture toughness. Toughness is a major challenge for polybenzoxazines, as they are prone to crack initiation and propagation. To overcome this limitation, various toughening strategies have been explored, including the incorporation of flexible segments, rubber particles, thermoplastics, and nanofillers such as graphene and carbon nanotubes. These additives improve energy dissipation through mechanisms such as crack deflection, crack bridging, and filler pull-out, thereby enhancing fracture and impact resistance [5,21]. However, conventional toughening agents often require high loadings, which can reduce strength and glass transition temperature. Fiber reinforcement further improves mechanical performance by increasing stiffness, strength, and impact resistance, making polybenzoxazine composites attractive for demanding structural applications. Nevertheless, achieving an optimal balance between stiffness, strength, and toughness remains a key objective in the design of high-performance polybenzoxazine materials.

### 4.3. Electrical Properties

Electrical properties such as dielectric constant, dielectric loss, conductivity, and breakdown strength are important for polybenzoxazine applications in electronics, energy storage, and electromagnetic interference (EMI) shielding. Polybenzoxazines are attractive dielectric materials because of their low moisture absorption, high purity, and tunable molecular structure. Typically, polybenzoxazines exhibit low dielectric constants (2.8–3.5) and low dielectric losses, making them suitable for high-frequency electronic applications. Molecular design strategies, including the incorporation of fluorinated, siloxane, or bulky substituents, can further reduce dielectric constant and loss by lowering polarizability and increasing free volume. For example, siloxane-containing polybenzoxazines have achieved dielectric constants as low as 2.67–2.82 with very low dielectric loss [32,33]. Although inherently insulating, polybenzoxazines can be made electrically conductive through the addition of carbon-based or metallic fillers, enabling applications in conductive composites and EMI shielding. Their combination of low dielectric loss, thermal stability, and environmental resistance makes polybenzoxazine composites promising materials for advanced electronic and electrical systems.

### 4.4. Chemical Resistance and Durability

Chemical resistance and durability are key advantages of polybenzoxazines, making them suitable for aerospace, marine, infrastructure, and chemical processing applications. Their highly crosslinked aromatic structure, low moisture uptake, and absence of hydrolyzable groups provide excellent resistance to solvents, water, chemicals, and environmental degradation. Polybenzoxazines exhibit low water absorption (typically <1%), high hydrolytic stability, and good resistance to acids, bases, and organic solvents. Their strong network structure also contributes to excellent dimensional stability and retention of electrical and mechanical properties in harsh environments. In addition, polybenzoxazines possess inherent flame retardancy due to their high char-forming ability, with limiting oxygen index (LOI) values typically between 30 and 40% [31,32,33,34,35,36]. Their combination of chemical resistance, thermal stability, and low moisture sensitivity supports long-term durability in demanding service conditions, while also enabling applications such as anticorrosion coatings, oil–water separation materials, and chemical-resistant adhesives. 

### 4.5. Wave-Transparent Properties

Research on the wave-transparent (radome) properties of benzoxazine resin composites has advanced rapidly in recent years, driven by the demand for high-speed aircraft, hypersonic vehicles, and missile radomes operating under high-temperature environments. Compared with conventional epoxy resins, polybenzoxazines exhibit inherently low dielectric constant (ε_r_ ≈ 2.6–3.2), low dielectric loss (tanδ < 0.01), high glass transition temperature (>200 °C), low moisture absorption, and excellent thermal stability, enabling stable electromagnetic transmission while maintaining mechanical integrity at elevated temperatures [80,81,82]. Consequently, benzoxazine resins have emerged as promising matrices for next-generation wave-transparent composites. Current research has focused on two main strategies. The first is the molecular design of low-dielectric benzoxazine resins, achieved by introducing fluorinated groups, siloxane segments, or bulky rigid structures to reduce molecular polarization and dielectric loss while preserving thermal resistance. The second is the fabrication of fiber-reinforced wave-transparent composites, particularly those reinforced with quartz fibers, which possess a dielectric constant of approximately 3.7–3.9 and excellent thermal resistance. Quartz fiber/polybenzoxazine composites combine the low dielectric characteristics of both constituents with high specific strength and outstanding dimensional stability, making them attractive candidates for radomes and other electromagnetic windows [35]. An important recent trend is the development of high-temperature wave-transparent composites. Unlike epoxy-based systems, whose dielectric and mechanical properties deteriorate near or above their glass transition temperature, quartz fiber/polybenzoxazine composites maintain relatively stable dielectric properties and structural integrity at temperatures exceeding 200 °C because of the highly crosslinked aromatic network and high char yield of benzoxazine. To further extend service temperatures, researchers have incorporated ceramic phases (e.g., SiO_2_ and h-BN), developed ceramizable benzoxazine composites, and combined benzoxazine with cyanate ester or phthalonitrile matrices. These approaches improve oxidation resistance, ablation resistance, and dielectric stability during prolonged high-temperature exposure, making them promising for reusable aerospace thermal protection systems requiring simultaneous load-bearing capability, thermal resistance, and electromagnetic transparency.

## 5. Processing Techniques and Morphological Control

Processing methods for polybenzoxazine composites must achieve complete curing and optimal network formation while maintaining practical manufacturing efficiency. Common techniques include compression molding, filament winding, autoclave processing, and resin transfer molding (RTM), all of which benefit from the low viscosity and low shrinkage of benzoxazine monomers. These methods enable good fiber impregnation, controlled fiber volume fractions, and low void content, making them suitable for high-performance composite fabrication. Cure kinetics play a central role in processing, as benzoxazine ring-opening polymerization is thermally activated and autocatalytic. Optimized curing and post-curing schedules are essential to maximize conversion, crosslink density, thermal stability, and glass transition temperature, with T_g_ values above 350 °C reported for advanced systems [13]. Emerging approaches such as additive manufacturing (3D printing) of polybenzoxazine vitrimers offer new opportunities for producing complex and recyclable components.

**Figure 9:**
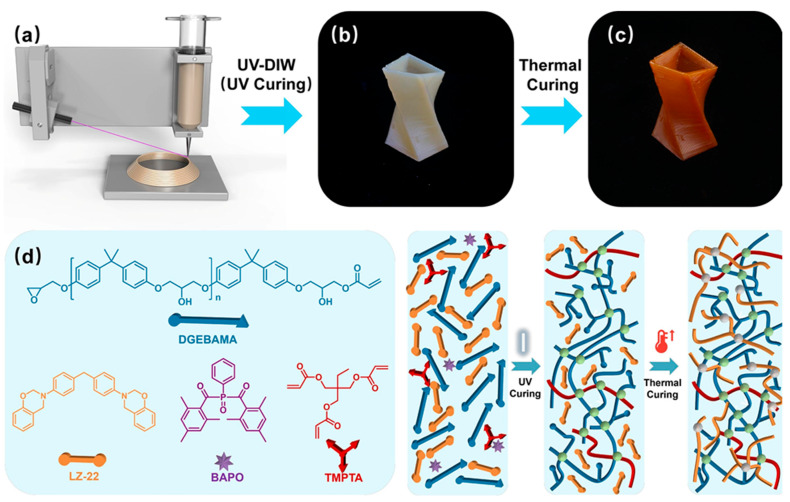
UV-assisted DIW 3D printing of BZs: (**a**) General route of UV-assisted DIW 3D printing; (**b**) 3D structure after UV curing; (**c**) 3D structure after thermal curing; and (**d**) chemical reactions occurring in BZs during the entire process. Reproduced with permission from [14].

Additive manufacturing (3D printing) has emerged as a powerful fabrication technology capable of producing complex geometries with high precision for applications ranging from transportation and biomedicine to electronics and aerospace. However, many current 4D-printing materials rely on acrylates and reactive diluents, which often compromise thermal and mechanical performance, limiting their use in demanding applications [83,84,85]. To address this challenge, Zhou et al. [14] developed dual-cure DIW 4D-printing inks based on benzoxazine and epoxy chemistry (Figure 9). DGEBAMA was synthesized from bisphenol A diglycidyl ether and acrylic acid, with residual acrylic acid catalyzing the ring-opening reactions of epoxy and benzoxazine during curing. The photocurable component enabled rapid printing of complex structures, while subsequent thermal curing produced isotropic networks with enhanced toughness and thermal stability. The copolymerized benzoxazine/epoxy network endowed BZ50 with excellent shape-memory behavior, combining high recovery, repeatability, and toughness through deformable ether linkages and a dense crosslinked structure. Compared with previously reported benzoxazine-based 3D printing materials, BZ50 exhibited superior overall performance, achieving a high T_g_ of 188 °C, tensile strength of 90 MPa, elongation at break of 9.34%, and excellent shape-memory behavior. This work demonstrates the potential of benzoxazine/epoxy systems as high-performance materials for advanced 4D-printing applications.

**Figure 10:**
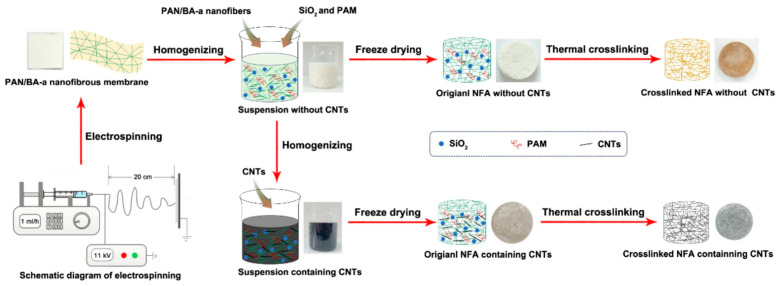
Schematic illustration of the fabrication process of polyacrylonitrile/benzoxazine nanofibrous aerogels (NFAs). Reproduced with permission from [86].

Zhou et al. [86] developed lightweight electrospun polyacrylonitrile/benzoxazine nanofibrous aerogels (NFAs) reinforced with carbon nanotubes (CNTs) as supports for shape-stabilized phase change materials (PCMs) (Figure 10). In the composite NFAs, electrospun PAN/BA-a nanofibers and SiO_2_ form a hierarchical 3D porous skeleton that enhances interfacial bonding through hydrogen interactions and provides multi-scale porosity. Upon heating, BA-a undergoes catalyst-free, by-product-free ring-opening polymerization to form a highly crosslinked polybenzoxazine network, which acts as a molecular glue to reinforce and stabilize the aerogel structure. CNTs further improve thermal conductivity and mechanical strength by bridging the porous network and providing multi-scale reinforcement. The highly porous aerogel structure enabled exceptionally high paraffin wax loading (up to 98 wt.%) without leakage while maintaining excellent thermal energy storage capacity and reliability. Pure PW (paraffin wax) exhibited onset and final decomposition temperatures of 255.3 °C and 320.4 °C, respectively, while PW/NFA-6 increased these values by 20.3 °C and 27.6 °C, indicating enhanced thermal stability. The thermal conductivity of the composite PCMs increased with CNT content, although the improvement was limited at low CNT loadings (<0.60 wt.%) due to the high PW fraction. At higher CNT contents (1.62–2.40 wt.%), a continuous thermal conduction network formed, enabling PW/NFA-6 to achieve a 125.3% increase in thermal conductivity compared with pure PW [87,88]. These composites show strong potential for thermal energy storage and thermal management applications.

Process optimization requires accurate monitoring of crosslinking during curing. As with conventional thermosets, vitrimer curing can generate thermal gradients and temperature overshoots, particularly in thick composites, due to exothermic reactions and low through-thickness thermal conductivity. These effects can lead to non-uniform curing, residual stresses, microcracking, and dimensional distortion. In addition, excessive curing temperatures may cause material degradation and reduce glass transition temperature, while incomplete curing can compromise mechanical performance, durability, and environmental resistance [89,90,91,92]. Overall, processing conditions strongly influence network morphology, filler dispersion, fiber orientation, and defect formation, which ultimately determine the thermal, mechanical, and functional performance of polybenzoxazine composites. Table 1 lists the properties of polybenzoxazine composites used in advanced applications and Table 2 compares the properties of different resin composite materials.

## 6. Applications

Polybenzoxazine composites have been demonstrated and proposed for diverse applications spanning aerospace structures, high-frequency electronics, protective coatings, energy storage, and emerging functional materials. The unique combination of high thermal stability, low polymerization shrinkage, excellent adhesion, flame retardancy, and molecular design flexibility enables polybenzoxazines to address performance requirements that are challenging for conventional thermosetting polymers. This section surveys key application areas and the specific property requirements that polybenzoxazines fulfill.

Aerospace applications represent a primary target for polybenzoxazine composites due to the demanding requirements for high-temperature performance, low flammability, structural efficiency, and long-term durability. Shen’s pioneering work demonstrated carbon fiber-reinforced polybenzoxazine composites with glass transition temperatures above 350 °C, moduli approximately 80% higher than comparable polyimide composites, and char yields of 87–92%, establishing the potential for aerospace structural applications. The high glass transition temperature enables service at elevated temperatures encountered in supersonic flight or near engines. The exceptional char yield and inherent flame retardancy address fire safety requirements for aircraft interiors and structures. The low polymerization shrinkage enables fabrication of large, complex structures with minimal residual stress and dimensional distortion. Current aerospace applications of polybenzoxazines remain limited compared to epoxies and bismaleimides, primarily due to concerns about brittleness and the need for extensive qualification testing, but ongoing development of toughened formulations and accumulation of long-term performance data are addressing these barriers.

High-frequency electronics applications leverage the low dielectric constant and low dielectric loss achievable through molecular design of polybenzoxazines. The increasing frequencies of wireless communication systems (5G and beyond) and high-speed digital circuits require substrate and packaging materials with low dielectric constants to minimize signal delay and crosstalk, and low dielectric loss to minimize signal attenuation and power dissipation. Yuan et al. demonstrated a siloxane-containing polybenzoxazine with tert-butyl substituents achieving a dielectric constant of 2.44 and dielectric loss of 0.0053 at 10 GHz, representing state-of-the-art performance for thermosetting polymers. Fluorinated benzoxazines have also been developed for low-k applications, with tailored solubility and reduced dielectric properties suitable for advanced circuit board materials. The combination of low dielectric properties, high thermal stability, low moisture absorption, and excellent adhesion to copper makes polybenzoxazines attractive for next-generation high-frequency circuit boards, chip packaging, and antenna substrates. Commercial adoption requires demonstration of reliability under thermal cycling, moisture exposure, and electrical stress, as well as compatibility with standard electronics manufacturing processes.

Protective coatings applications exploit the excellent adhesion, chemical resistance, and thermal stability of polybenzoxazines. The phenolic hydroxyl groups in cured polybenzoxazines provide strong adhesion to metal, ceramic, and polymer substrates through hydrogen bonding and chemical interactions. The highly crosslinked aromatic structure provides excellent resistance to solvents, acids, bases, and environmental degradation. Bio-based polybenzoxazines have been demonstrated for anticorrosion coatings, with specific corrosion-resistant formulations reported for cardanol and eugenol polybenzoxazine systems. The inherent flame retardancy and low smoke generation make polybenzoxazine coatings attractive for fire protection applications in aerospace, transportation, and buildings. The low polymerization shrinkage minimizes coating stress and improves adhesion durability. Applications include corrosion-resistant coatings for marine and chemical processing equipment, fire-resistant coatings for structural steel, and protective coatings for electronic components. Bio-based polybenzoxazine composites have been demonstrated for oil-water separation, exploiting the inherent hydrophobicity of the polybenzoxazine network combined with porous structures that allow selective permeation. The chemical resistance enables use in harsh chemical environments where conventional polymer membranes would degrade. The thermal stability enables high-temperature filtration and sterilization. The development of polybenzoxazine membranes with controlled pore size, surface chemistry, and mechanical properties represents an emerging application area with potential for water treatment, chemical separations, and gas separations.

Energy storage applications including supercapacitors and battery components have been proposed for polybenzoxazine composites, though comprehensive device-level performance data is limited in the supplied literature. The combination of thermal stability, chemical resistance, and the ability to incorporate conductive fillers makes polybenzoxazines attractive for electrode binders, separators, and packaging in energy storage devices. The low moisture absorption is advantageous for preventing electrolyte degradation and maintaining performance in humid environments. The development of polybenzoxazine-based energy storage materials requires demonstration of electrochemical stability, ionic conductivity (for separators), and long-term cycling stability, with explicit, reproducible supercapacitor performance metrics not consistently presented across the supplied literature.

Adhesive applications exploit the excellent adhesion of polybenzoxazines to diverse substrates combined with high-temperature performance and chemical resistance. The phenolic hydroxyl groups provide strong adhesion to metals, ceramics, and polymers, while the low polymerization shrinkage minimizes adhesive stress and improves joint durability. High-temperature structural adhesives based on polybenzoxazines have been developed for aerospace and automotive applications requiring service temperatures above 200 °C. The chemical resistance enables use in harsh environments where epoxy adhesives would degrade. The development of toughened polybenzoxazine adhesives with improved peel strength and impact resistance represents an important direction for expanding adhesive applications.

## 7. Challenges and Future Perspectives

Despite the considerable progress and broad application potential of polybenzoxazine composites, several challenges continue to hinder their widespread commercial adoption. Key issues include network brittleness, high curing temperatures, limited recyclability, scalability constraints, and insufficient characterization standards. At the same time, emerging developments such as dynamic covalent networks, vitrimer chemistry, self-healing systems, and advanced computational design offer promising solutions. One of the primary limitations of polybenzoxazines is their inherent brittleness. The highly crosslinked aromatic network responsible for excellent thermal stability and high glass transition temperatures also results in low fracture toughness and impact resistance. Conventional toughening approaches, including rubber modification and thermoplastic incorporation, often compromise thermal performance. Bio-based benzoxazines derived from cardanol or eugenol provide improved toughness through flexible side chains, while nanofillers such as graphene and carbon nanotubes enhance crack deflection and bridging mechanisms. Future work should focus on achieving toughness levels comparable to advanced epoxy systems without sacrificing thermal and chemical resistance.

High curing temperatures, typically above 200 °C, increase processing costs and limit substrate compatibility. Although catalysts can reduce cure temperatures, they may negatively affect storage stability or introduce impurities. Therefore, developing low-temperature-curable benzoxazine systems (<150 °C) and photocurable formulations remains a major research priority. Sustainability and end-of-life management are also critical concerns. Conventional polybenzoxazines are difficult to recycle because of their permanently crosslinked structure. Dynamic covalent chemistries, including disulfide, imine, and transesterification linkages, have enabled the development of recyclable polybenzoxazine vitrimers with self-healing and reprocessing capabilities. Future efforts should optimize the balance between recyclability and high-temperature performance while demonstrating long-term property retention over multiple recycling cycles. Self-healing functionality, enabled by reversible bond exchange reactions, represents another attractive feature of dynamic polybenzoxazines. Such materials can repair microcracks and surface damage under external stimuli, extending service life and reliability. However, further studies are required to demonstrate effective healing in fiber-reinforced composites under realistic operating conditions.

Commercial implementation also depends on overcoming manufacturing challenges related to monomer cost, formulation stability, process reproducibility, and compatibility with existing composite-processing technologies. The development of scalable synthesis routes, latent one-part formulations, and low-temperature curing systems will be crucial for industrial adoption. Another limitation is the lack of standardized characterization protocols. Critical properties such as fracture toughness, fatigue resistance, environmental durability, recyclability, EMI shielding effectiveness, and electrochemical performance are often reported inconsistently, making direct comparison difficult. Standardized testing and comprehensive structure–property datasets are needed to support predictive materials design. Computational modeling and machine learning are emerging as powerful tools for accelerating polybenzoxazine development. Given the vast molecular design space, data-driven approaches can help predict material properties, identify promising formulations, and optimize composite performance, reducing reliance on extensive experimental screening.

The growing interest in bio-based polybenzoxazines further supports sustainability goals. Renewable feedstocks such as cardanol, eugenol, vanillin, and lignin-derived phenols, together with solvent-free synthesis routes and bio-based fillers, offer environmentally friendly alternatives to petroleum-derived systems. Future studies should focus on life-cycle assessment, performance optimization, and feedstock consistency. Finally, multifunctional polybenzoxazine composites capable of simultaneously providing structural integrity, thermal management, electrical conductivity, sensing, and energy-storage functions represent a rapidly expanding research area. The integration of advanced fillers, hierarchical architectures, and stimuli-responsive designs is expected to drive the development of next-generation high-performance multifunctional materials.

## 8. Conclusions

Polybenzoxazines are a versatile class of high-performance thermosetting polymers that combine excellent thermal stability, low curing shrinkage, strong adhesion, flame resistance, and exceptional molecular design flexibility. This review has highlighted the structure–property relationships governing polybenzoxazine composites, from monomer design and network formation to reinforcement strategies, processing methods, and advanced applications. The tunable chemistry of benzoxazines enables precise control over thermal, mechanical, electrical, and environmental properties. Through molecular engineering, glass transition temperatures above 350 °C, dielectric constants as low as 2.67, and enhanced toughness have been achieved. Their ring-opening polymerization proceeds with minimal shrinkage and without volatile byproducts, facilitating the fabrication of dimensionally stable, low-void composites. Significant performance improvements have been realized through the incorporation of carbon nanomaterials, fiber reinforcements, and hybrid fillers. Carbon fiber-reinforced polybenzoxazines exhibit outstanding thermal stability and mechanical performance, while bio-based benzoxazines combined with functional nanofillers provide enhanced toughness, flame retardancy, and sustainability. These advances have expanded applications in aerospace, electronics, coatings, membranes, and energy-related systems.

Despite these advantages, challenges such as brittleness, high curing temperatures, limited recyclability, and manufacturing scalability continue to hinder broader industrial adoption. Emerging strategies, including dynamic covalent networks, vitrimer chemistry, self-healing systems, and low-temperature curing formulations, offer promising solutions while improving sustainability. Future research should focus on tougher, recyclable polybenzoxazines, scalable manufacturing, standardized characterization, and computationally guided materials design. Advances in bio-based formulations and multifunctional composites are expected to further expand their applications, reinforcing polybenzoxazines as a leading platform for high-performance thermosetting composites in aerospace, electronics, energy, and infrastructure.

## Figures and Tables

**Table 1:** Properties of polybenzoxazine composites used in advanced applications.

Material	Bzo Monomer	Properties	Application	Reference
PBZ/RGO-CS	BA-a type benzoxazine (from bisphenol A and aniline)	Excellent separation efficiency (99.1%) High oil absorption capacity (66–123 g/g)	Efficient oil–water separation	[53]
P-AE-MV-CF	Bio-Benzoxazine synthesized from vanillin and 1,10-diaminodecane	Low activation energy for bond exchange (E_a_ = 48.1 kJ mol^−1^) Self-healing efficiency (98.2% at 160 °C)	Recyclable CFRPs	[1]
p(HBA-ole-pd)-CF composite	Benzoxazine synthesized from 4-hydroxybenzaldehyde and oleylamine	Lower activation energy for imine exchange (61.35 kJ mol^−1^) Faster stress relaxation (50 s at 140 °C)	High-performance recyclable composites	[56]
BN@2Cu/PBz composites	Benzoxazine resin, AIBZ682	Thermal conductivity (1.049 W m^−1^ K^−1^)	Thermal management applications	[74]
POSS-CFRP (PBZ_10_)	Araldite^®^ MT35700 and Araldite^®^ BDP4903 (liquid mono-benzoxazine)	Best retention of glass transition temperature, flexural strength, and stiffness after up to 18 months of simulated exposure.	Durable deployable space structures	[57]
BZ50	DDM-Ph-type benzoxazine Resin (LZ-22)	Tensile strength (92 MPa), Elongation at break (9.3%), Excellent shape-memory behavior	DIW 4D-printing inks	[14]
PW/NFA composite	BA-a benzoxazine monomer	High paraffin wax loading (up to 98 wt.%) without leakage	Shape-stabilized phase change materials	[83]

Benzoxazine resin, AIBZ682 was purchased from ACO Pharm Co., Ltd., Shanghai, China; Araldite^®^ MT35700 and Araldite^®^ BDP4903 were purchased from Huntsman Advanced Materials Ltd., Basel, Switzerland.

**Table 2:** Comparative Performance of Different Resin Composite Materials.

Resin Matrix	T_g_ (°C)	Service Temperature (°C)	Dielectric Constant (10 GHz)	Dielectric Loss (×10^−3^)	Char Yield (%)	Ablation Resistance	Mechanical Properties	Reference
Polybenzoxazine (PBz)	180–300	200–300	2.5–3.2	2–8	40–65	Excellent	High strength and toughness; low shrinkage	[80,93]
Phenolic resin (PF)	150–200	180–250	3.5–5.0	10–30	55–70	Excellent	Moderate; relatively brittle	[94]
Arylacetylene resin (AA)	300–400	300–350	2.7–3.2	2–8	65–80	Excellent	High modulus and strength	[95,96]
Phthalonitrile resin (PN)	350–450	350–400	2.8–3.4	2–8	70–85	Excellent	Excellent at elevated temperature	[97]
Bismaleimide (BMI)	220–300	220–280	3.0–3.5	5–15	35–50	Good	High strength and stiffness	[98]
Cyanate ester (CE)	250–350	250–300	2.6–3.0	1–5	35–55	Good	Excellent dimensional stability	[99]
Epoxy resin (EP)	120–220	120–180	3.2–4.2	10–25	10–30	Poor-Moderate	Excellent room temperature properties	[100]

## Data Availability

No new data were created or analyzed in this study. Data sharing is not applicable to this article.
